# Draft Genome Sequence of *Coprobacter fastidiosus* NSB1^T^

**DOI:** 10.1128/genomeA.00122-14

**Published:** 2014-03-06

**Authors:** A. V. Chaplin, B. A. Efimov, E. V. Khokhlova, L. I. Kafarskaia, A. E. Tupikin, M. R. Kabilov, A. N. Shkoporov

**Affiliations:** a Pirogov Russian National Research Medical University, Moscow, Russia; b Institute of Chemical Biology and Fundamental Medicine SB RAS, Novosibirsk, Russia; c SB RAS Genomics Core Facility, Novosibirsk, Russia

## Abstract

*Coprobacter fastidiosus* is a Gram-negative obligate anaerobic bacterium belonging to the phylum *Bacteroidetes*. In this work, we report the draft genome sequence of *C. fastidiosus* strain NSB1^T^ isolated from human infant feces.

## GENOME ANNOUNCEMENT

The human intestine is colonized by a large and diverse community of microorganisms that exert protective, structural, and metabolic effects on the intestinal mucosa and other systems of the human host (1). The majority of these microbes belong to the bacterial phyla *Firmicutes* and *Bacteroidetes* (2). Species of the latter group account for about 50% of the 16S rRNA gene sequences detected in human fecal samples (3) and are considered to play a significant role in the utilization of complex carbohydrates and the regulation of mucosal immunity (4). At the same time, certain members of the *Bacteroidetes* group can cause various opportunistic infections (5).

The genus *Coprobacter*, described in 2013, is classified within the family *Porphyromonadaceae*, the order *Bacteroidales*, and the phylum *Bacteroidetes*. Currently, it comprises a single species, *Coprobacter fastidiosus*, isolated from the feces of a healthy infant (6). These Gram-negative obligate anaerobic bacteria differ from members of a closely related genus, *Barnesiella*, by their ability to produce propionic acid, their lower G+C content, and the composition of their major fatty acids. The abundance of 16S rRNA sequences of *C. fastidiosus* in metagenomic studies suggests this species is highly prevalent in human intestinal microbiota (6).

A paired-end library of *C. fastidiosus* NSB1^T^ was made using a Nextera DNA sample preparation kit (Illumina). The library was quality checked using the Agilent high sensitivity DNA kit and the 2100 Bioanalyzer (Agilent Technologies). Sequencing was performed on a MiSeq benchtop sequencer using the MiSeq reagent kit version 1, with a read length of 150 bases on each strand.

The reads were assembled *de novo* using the CLC Genomics Workbench 6.0, with a word size of 19 and a bubble size of 143. The overall length of the contigs is 3.4 Mb, the N_50_ is 167 kb, and the G+С content is 38.3%. The genome was annotated using the NCBI Prokaryotic Genome Annotation Pipeline (7). Metabolic pathways were reconstructed using KAAS (8) and manually curated.

The predicted metabolic pathways are typical for chemoorganoheterotrophic bacteria. An analysis of the core metabolism genes suggests the presence of a Wood-Werkman cycle, a partial tricarboxylic acid (TCA) cycle lacking a succinyl-coenzyme A (CoA) hydrolase, as previously described for *Propionibacterium freudenreichii*, and a respiratory chain that can be used in the production of propionic acid (9). Acetyl-CoA is likely synthesized by pyruvate-flavodoxin oxidoreductase (gene NSB1T_03985). The presence of the genes coding for cytochrome *bd* oxidase (NSB1T_14095 and NSB1T_14100) may be indicative of the ability of the strain to utilize oxygen at low concentrations for energy production (10, 11).

No prophage sequences were found using PHAST (12). The resistance of *C. fastidiosus* NSB1^T^ to β-lactam antibiotics (6) was linked to the presence of several possible β-lactamase genes (NSB1T_03925, NSB1T_10950, NSB1T_07185, and NSB1T_06290). However, despite the presence of a predicted chloramphenicol acetyltransferase gene (NSB1T_01810), the strain NSB1^T^ is sensitive to chloramphenicol (6).

The availability of this genomic sequence makes possible further studies to reveal molecular factors allowing for the colonization of human gut by *C. fastidiosus*, as well as to understand the functional position of this recently discovered species within the human intestinal microbial consortium.

### Nucleotide sequence accession numbers.

This whole-genome shotgun project has been deposited to DDBJ/EMBL/GenBank under the accession no. AWWG00000000. The version described in this paper is the first version, AWWG01000000.
